# Effects of high- and low-yield moso bamboo (*Phyllostachys edulis*) forests on bacterial community structure

**DOI:** 10.1038/s41598-023-36979-4

**Published:** 2023-06-17

**Authors:** Fang Liu, Zong-sheng Yuan, Zhi-hao Zeng, Hui Pan

**Affiliations:** 1 College of Life Sciences, Fujian Agriculture and Forestry University, Fuzhou, 350002 Fujian China; 2 Institute of Oceanography, Minjiang University, Minhou County, Fuzhou, 350108 Fujian China

**Keywords:** Ecology, Microbiology

## Abstract

To study the characteristics of bacterial community structure in high-yield and low-yield moso bamboo (*Phyllostachys edulis*) forests, we collected bamboo rhizome, rhizome root, stem, leaf, rhizosphere soil, and non-rhizosphere soil from high- and low-yield forests in Yong'an City and Jiangle County of Fujian Province, China. The genomic DNA of the samples was extracted, sequenced and analyzed. The results show that: the common differences between the high-yield and low-yield *P. edulis* forest samples in the two regions were mainly in bacterial community compositions in the bamboo rhizome, rhizome root, and soil samples. Differences in the bacterial community compositions in the stem and leaf samples were insignificant. The bacterial species and diversity in rhizome root and rhizosphere soil of high-yield *P. edulis* forests were less than those of low-yield forests. The relative abundance of Actinobacteria and Acidobacteria in rhizome root samples of high-yield forests was higher than that in low-yield forests. The relative abundance of Rhizobiales and Burkholderiales in bamboo rhizome samples in high-yield forests was higher than that in low-yield forests. The relative abundance of Bradyrhizobium in bamboo rhizome samples in high-yield forests was higher than that in low-yield forests in the two regions. The change of bacterial community composition in *P. edulis* stems and leaves showed little correlation with high- or low-yields of *P. edulis* forests. Notably, the bacterial community composition of the rhizome root system was correlated with the high yield of bamboo. This study provides a theoretical basis for using of microbes to enhance the yields of *P. edulis* forests.

## Introduction

Bamboo is widely distributed in Asia, Africa, and Latin America. Existing statistics indicate that there are more than 1200 bamboo species worldwide, accounting for about 3.2% of the total forest area^1–3^. Moso bamboo (*Phyllostachys edulis*) is the bamboo species with the highest economic value and is the most widely distributed among the bamboo. It grows fast and is high yielding. It is thus an important bamboo raw material, which also produces by-products, such as bamboo shoots^4^. The yield of *P. edulis* forest directly affects its economic benefits^5,6^. According to the difference in the yield of *P. edulis* forests, they can be divided into high-yield forest and low-yield forest. The standing bamboo density of high-yield *P. edulis* forests is generally more than 1.5 times that of low-yield forests^7^. The yield of single-stage bamboo (every 2 years) and bamboo shoots in the high-yield forest was more than twice that of the low-yield forest^7^. The average DBH of bamboo stems was also larger than that of low-yielding forests^8^. The soil quality, water supply and climatic conditions of the high-yield *P. edulis* forest are relatively superior to those of the low-yield forest^9^. The most important factor is soil quality^10^. High soil quality can make bamboo get more nutrients and be less affected by diseases and insect pests^11^. However, the internal mechanism leading to the soil quality of *P. edulis* is less studied at present. Studying the differences between high-yield and low-yield forests and the formation mechanisms of high-yield *P. edulis* forests is thus of great significance in reforming the low-yield *P. edulis* forests and improving their economic benefits.

Soil quality is closely related to the soil microbiome^12^. The rhizosphere is the interface of material exchange between plants and soil ecosystems and is also the area where soil microorganisms are enriched^13,14^. Its core microbial community and structure are closely related to the nutrients in the soil and the disease-suppressing ability of plants. Plant surfaces and their internal tissues are enriched with large quantities of microorganisms^15,16^. Some of their types may play a beneficial role in reducing the presence of pathogens^17^. Microbial communities inside and outside plants are selected during plant growth and development, forming unique microbial communities^18,19^. And the rhizosphere microbial community structure of different plants is unique and representative^20,21^. These changes in microbial community structure affect plant growth and development^22,23^. Therefore, it is possible to preliminarily explore the internal mechanisms of high-yield and low-yield *P. edulis* forests by studying the differences between endophytic and rhizosphere microbial communities in high-yield forests and low-yield forests.

In this study, the differences and diversity of endophytic and rhizosphere bacterial community structure in the bamboo rhizome, rhizome root, stem, leaf, and soil from high-yield and low-yield *P. edulis* forests were studied using Illumina high-throughput sequencing technology^16^. The findings of this study lay a foundation for future research on the correlation between the high-yield cultivation of *P. edulis* forests and the bacterial microbiome.

## Results

### Analysis of sequencing results

**Figure 1:**
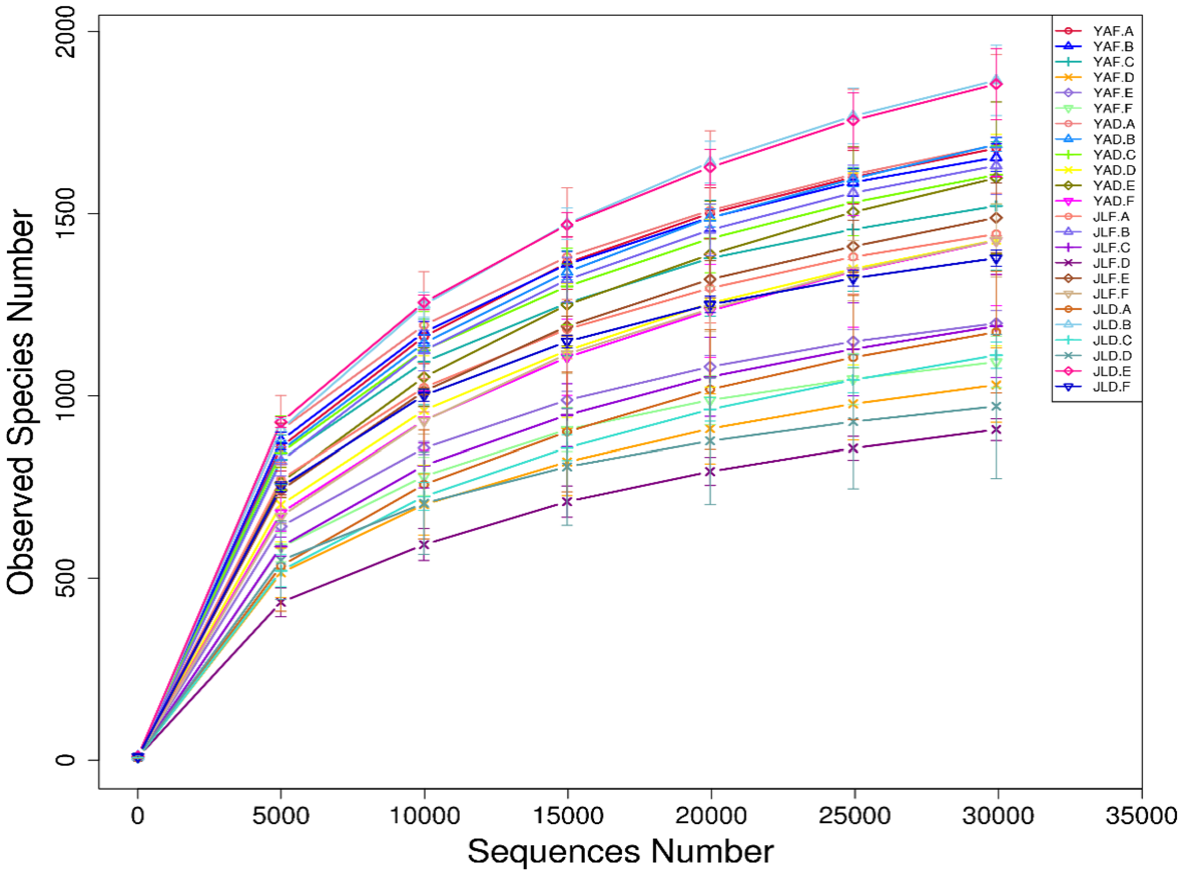
Dilution curves at 97% similarity level of high-yield and low-yield *P. edulis* forest samples in the two experimental sites (the specific information of the sample code is shown in Table 2, the same below).

A total of 5,745,495 valid sequences, with an average length of 377 bp, were obtained after high-throughput sequencing of 72 samples. The sequencing coverage of each sample was more than 99.8%, and the sequencing results were relatively consistent amongst the replications for each treatment. The slope of the dilution curve of each group decreased, tended to be flat and then plateaued with the increase of the sequencing amount (Fig. 1). This phenomenon indicates that only a small number of new OTUs are generated as the amount of sequencing increases. The sequencing volume tends to be saturated. In general, the amount of sequencing data in this study is reasonable, and it can reflect the status of the samples.

**Figure 2:**
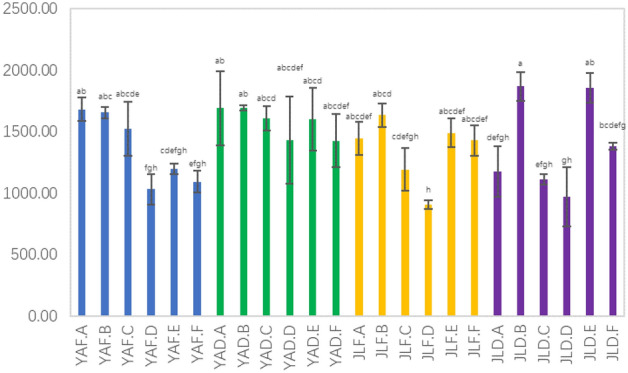
Observed bacterial species number of each sample.

It can be seen from the number of bacterial species observed in the samples in Fig. 2 that the number of species observed in the rhizosphere soil samples of the low-yield *P. edulis* forest in Jiangle is the largest, and it is significantly different from other samples. In the same area, the leaf samples of high-yielding *P. edulis* forest had the least number of species. The average number of species observations of most samples in Yong'an area was higher than that in Jiangle area, and generally along the direction of soil, roots, stems, and leaves, the number of species observations showed a gradual downward trend.

### Analysis of the bacterial community composition

**Figure 3:**
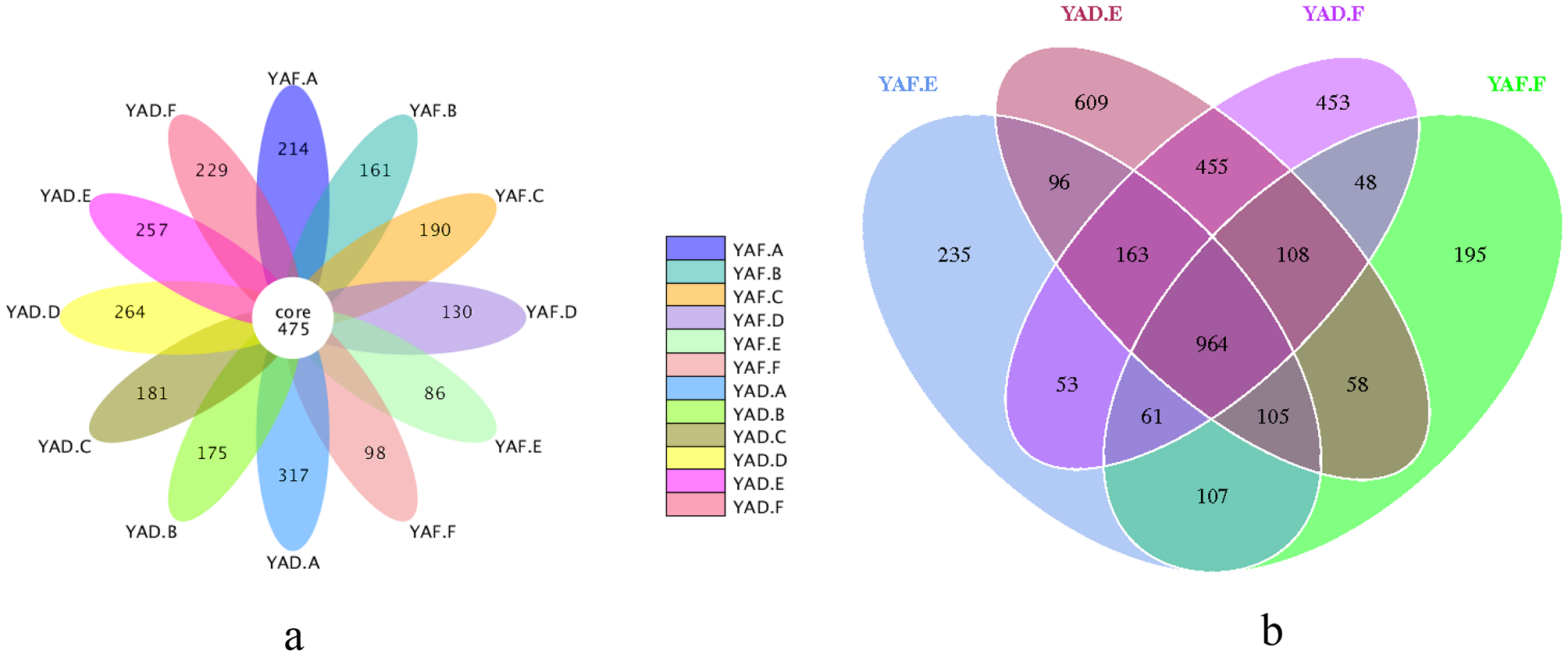
The number and distribution of OTUs in samples from high-yield and low-yield *P. edulis* forests in Yong'an. (**a**) Comparison of plant tissue samples; (**b**) Comparison of soil samples.

**Figure 4:**
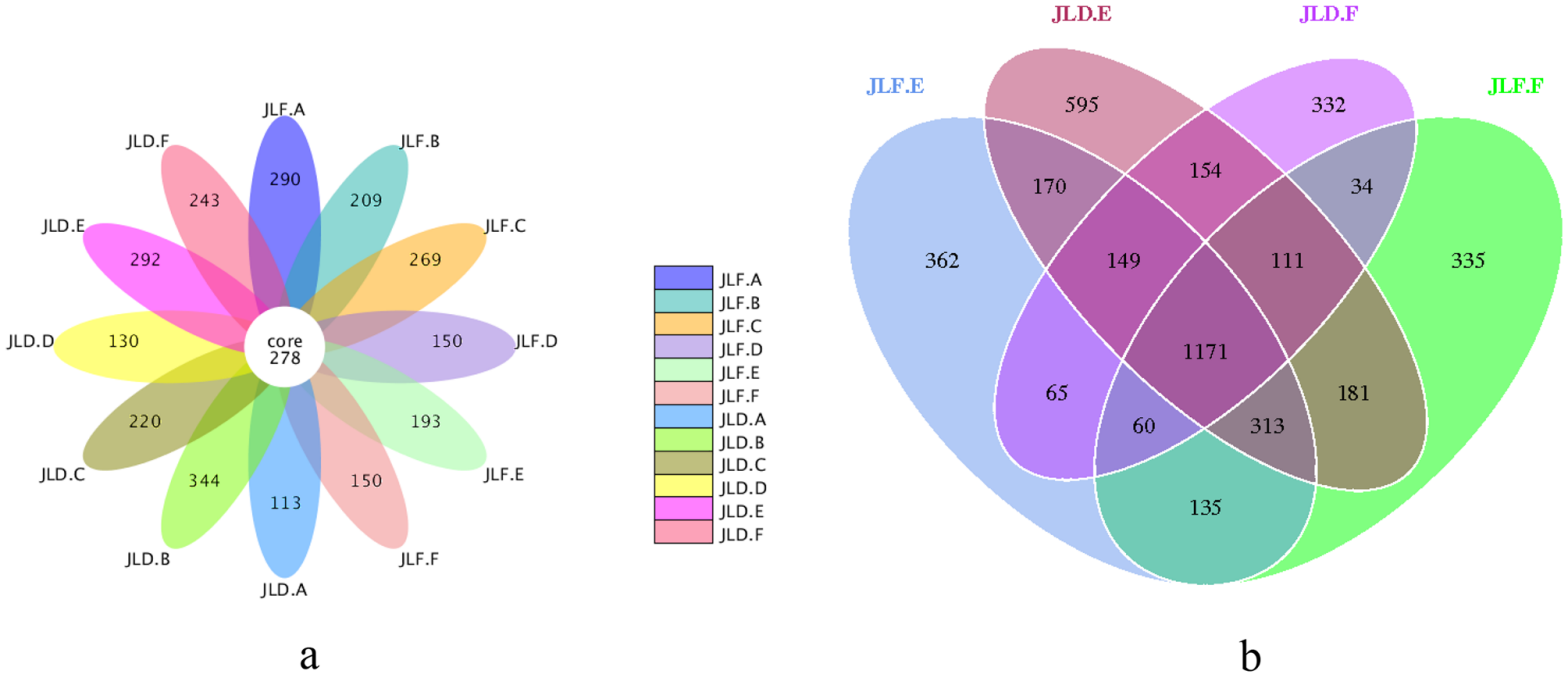
The number and distribution of OTUs in samples from high-yield and low-yield *P. edulis* forests in Jiangle.

A total of 57 phyla, 132 classes, 285 orders, 459 families, and 781 genera of bacteria were identified from the 72 samples sequenced. Figures 3 and 4 show the 475 common OTUs identified from the bamboo rhizome, rhizome root, stem, and leaf samples and 964 common OTUs identified from the soil samples collected from the high-yield and low-yield *P. edulis* forests in Yong'an. In Jiangle, there were 278 common OTUs identified from the bamboo rhizome, rhizome root, stem, and leaf samples, and 1171 common OTUs identified from the soil samples collected from the high-yield and low-yield forests. These data suggested that the number of common OTUs in the soil samples was 2–4 times that of the samples of bamboo tissues, indicating more species and greater bacterial diversity in the soil samples relative to those in the bamboo tissue samples. Notably, the rhizome root and rhizosphere soil samples in high-yield *P. edulis* forest displayed a much smaller number of unique OTUs than that of low-yield forest samples. This finding suggested the presence of fewer species and lower bacterial diversity in the rhizome root and rhizosphere soil of high-yield *P. edulis* forests than in the low-yield forest.

**Figure 5:**
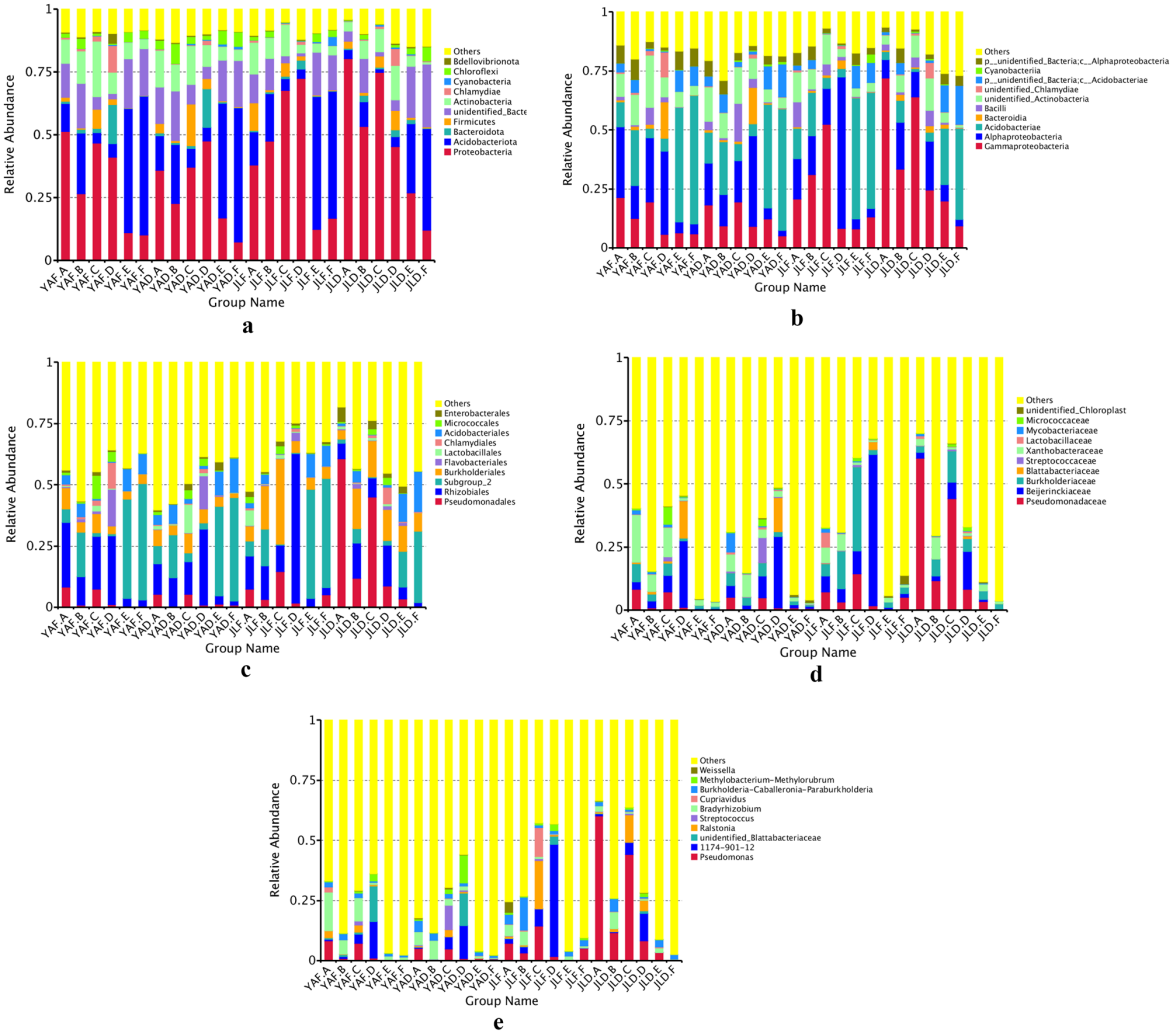
The relative abundance of bacterial communities at different levels in the high-yield and low-yield *P. edulis* forests in the two regions. (**a**) Phyla level; (**b**) class level; (**c**) order level; (**d**) family level; (**e**) genus level.

The sequences were classified into OTUs at a 97% similarity level, followed by the generation of a relative abundance histogram of the bacterial community after statistical analysis. Analysis was conducted at various classification levels to further understand the bacterial community structure of high-yield and low-yield *P. edulis* forests. The relative abundance of Actinobacteria (at the phyla level) in the rhizome root and stem samples in the high-yield *P. edulis* forest was higher than that in the low-yield forest (Fig. 5a). Similarly, the relative abundance of Acidobacteria in the soil samples of the high-yield *P. edulis* forest was higher than that in the low-yield forest. In contrast, the relative abundance of Proteobacteria exhibited an opposite trend.

The common feature of *P. edulis* forest samples from the two sites (at the class level) was that the relative abundance of α-Proteobacteria in bamboo rhizome samples in the high-yield forest was higher than that in the low-yield forest. Similarly, the relative abundance of Acidobacteria in soil samples from the high-yield forest was higher than that of the low-yield forest. In contrast, the relative abundance of γ-Proteobacteria in leaf and soil samples from the low-yield forest was higher than that in the high-yield forest (Fig. 5b). At the order level, the bamboo rhizome samples from the high-yield and low-yield forests in the two sites exhibited the same differences, but the relative abundance of Rhizobiales and Burkholderiales in the bamboo rhizome samples from the high-yield forest was higher than that from the low-yield forest (Fig. 5c). In the same line, the relative abundance of Burkholderiaceae and Flavobacteriaceae in bamboo rhizome samples from the high-yield forest was higher than that of the low-yield forest. Similarly, the relative abundance of Beijerinckiaceae in the rhizome root samples from the high-yield forest was higher than that from the low-yield forest (Fig. 5d). At the genus level (Fig. 5e), the relative abundance of Bradyrhizobium in the bamboo rhizome samples from the high-yield forest in the two sites was higher than that from the low-yield forest. Generally, the composition of bacterial communities in the bamboo rhizome, rhizome root, and soil samples of high-yield and low-yield forests in Yong'an and Jiangle showed consistent differences at all levels of classification, indicating that the bacterial community structure of the bamboo rhizome system in the forests has a certain correlation with the yield of *P. edulis* forests.

### Alpha diversity analysis of the bacterial community

**Figure 6:**
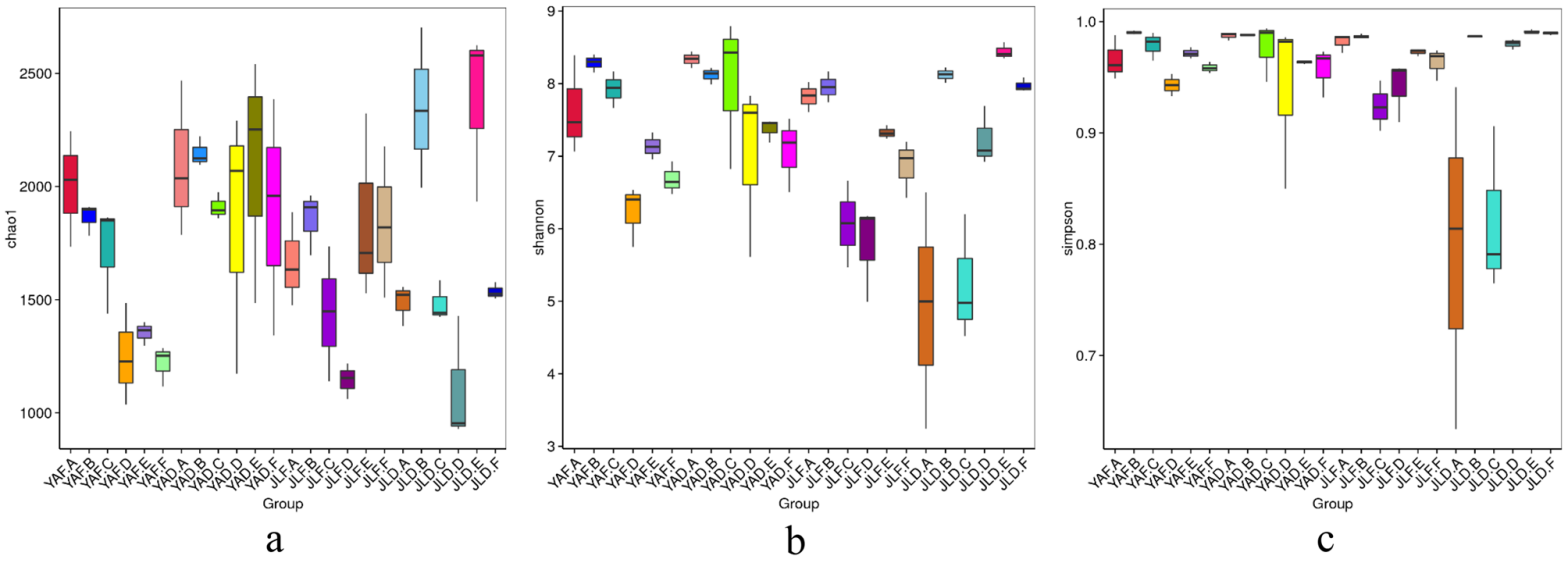
Box chart showing diversity index differences between high-yield and low-yield *P. edulis* forest samples in the two regions. (**a**) Chao1 index; (**b**) Shannon index; (**c**) Simpson index.

In Yong'an, the rhizome, leaf and soil samples of the low-yield Moso bamboo forest showed significantly higher chao1 index and similar values of Shannon and Simpson indexes compared to the high-yield forest (Table 1, Fig. 6). In Jiangle, the rhizome root, leaf and soil samples of the low-yield *P. edulis* forest displayed a significantly higher chao1 index (Table 1, Fig. 6a), whereas bamboo rhizome and stem samples of the high-yield forest had greater values of Shannon (Fig. 6b) and Simpson (Fig. 6c) indexes compared with the high-yield forest. However, lower values were recorded for leaf and soil samples compared to those of low-yield *P. edulis* forest.

**Table 1:** Alpha diversity index of high-yield and low-yield *P. edulis* forest samples in the two regions.

Group	Observed_species	Shannon	Simpson	Chao1	ACE	Goods_coverage
YAF.A	1679.33 ± 96.02ab	7.64 ± 0.68abc	0.97 ± 0.02a	2002.77 ± 256.41abcde	2036.59 ± 249.78abc	0.99 ± 0.00a
YAF.B	1654.33 ± 47.12abc	8.28 ± 0.12a	0.99 ± 0.00a	1863.97 ± 70.16abcdef	1912 ± 52.91abc	0.99 ± 0.00a
YAF.C	1521 ± 216.81abcde	7.92 ± 0.25a	0.98 ± 0.01a	1717.45 ± 241.46abcdef	1774.85 ± 215.42abc	0.99 ± 0.00a
YAF.D	1029.33 ± 125.3fgh	6.23 ± 0.42bcde	0.94 ± 0.01a	1249.74 ± 225.17def	1288.17 ± 163.6bc	0.99 ± 0.00a
YAF.E	1198.67 ± 42.67cdefgh	7.14 ± 0.19abcd	0.97 ± 0.01a	1353.66 ± 52.5cdef	1411.66 ± 59.66bc	0.99 ± 0.00a
YAF.F	1092.33 ± 86.23efgh	6.68 ± 0.23abcd	0.96 ± 0.01a	1218.03 ± 89.88def	1263.91 ± 94.64bc	0.99 ± 0.00a
YAD.A	1688.33 ± 304.13ab	8.33 ± 0.11a	0.99 ± 0.00a	2096.95 ± 344.75abcd	2080.46 ± 361.64ab	0.99 ± 0.00a
YAD.B	1690 ± 22.91ab	8.12 ± 0.11a	0.99 ± 0.00a	2147.6 ± 66.08abc	2173.8 ± 44.64ab	0.98 ± 0.00a
YAD.C	1606.33 ± 97.5abcd	8.01 ± 1.05a	0.98 ± 0.03a	1910.07 ± 58.94abcdef	1974.42 ± 31.46abc	0.99 ± 0.00a
YAD.D	1427.33 ± 355.36abcdef	7.01 ± 1.22abcd	0.94 ± 0.08a	1844.06 ± 591.65abcdef	1870.78 ± 634.58abc	0.99 ± 0.01a
YAD.E	1597.67 ± 256.14abcd	7.37 ± 0.16abcd	0.96 ± 0.00a	2092.83 ± 544.65abcd	2145.1 ± 572.69ab	0.98 ± 0.01a
YAD.F	1425.33 ± 217.81abcdef	7.07 ± 0.52abcd	0.96 ± 0.02a	1895.59 ± 525.01abcdef	1944.59 ± 519.15abc	0.98 ± 0.01a
JLF.A	1444 ± 137.23abcdef	7.82 ± 0.21ab	0.98 ± 0.01a	1665.21 ± 207.11abcdef	1722.56 ± 203.45abc	0.99 ± 0.00a
JLF.B	1631.67 ± 96.76abcd	7.95 ± 0.21a	0.99 ± 0.00a	1855.14 ± 139.77abcdef	1948.07 ± 130.11abc	0.99 ± 0.00a
JLF.C	1191 ± 173.70cdefgh	6.07 ± 0.60cde	0.92 ± 0.02a	1441.06 ± 298.36cdef	1473.6 ± 278.04bc	0.99 ± 0.00a
JLF.D	907 ± 36.59 h	5.77 ± 0.67de	0.94 ± 0.03a	1144.09 ± 78.47ef	1166.91 ± 31.45c	0.99 ± 0.00a
JLF.E	1488.67 ± 117.01abcdef	7.33 ± 0.09abcd	0.97 ± 0.00a	1852.24 ± 417.23abcdef	1904.7 ± 449.62abc	0.99 ± 0.01a
JLF.F	1426.33 ± 123.52abcdef	6.86 ± 0.40abcd	0.96 ± 0.01a	1835.67 ± 334.06abcdef	1885.42 ± 348.56abc	0.99 ± 0.00a
JLD.A	1174.33 ± 204.67defgh	4.91 ± 1.63e	0.8 ± 0.15b	1487.23 ± 91.2bcdef	1569.81 ± 89.72abc	0.99 ± 0.00a
JLD.B	1866 ± 118.19a	8.12 ± 0.10a	0.99 ± 0.00a	2344.77 ± 353.72ab	2387.77 ± 353.68a	0.98 ± 0.01a
JLD.C	1111 ± 44.23efgh	5.23 ± 0.87e	0.82 ± 0.08b	1483.58 ± 88.41bcdef	1528.29 ± 113.56abc	0.99 ± 0.00a
JLD.D	970.67 ± 242.16gh	7.23 ± 0.41abcd	0.98 ± 0.00a	1103.43 ± 281.16f.	1155.46 ± 317.75c	0.99 ± 0.00a
JLD.E	1855.67 ± 120.03ab	8.44 ± 0.11a	0.99 ± 0.00a	2379.31 ± 386.18a	2413.21 ± 376.81a	0.98 ± 0.01a
JLD.F	1377.67 ± 27.47bcdefg	7.97 ± 0.10a	0.99 ± 0.00a	1536.29 ± 36.82abcdef	1569.51 ± 31.99abc	0.99 ± 0.00a

The common characteristics of the high-yield and low-yield forest samples in the two regions were that the rhizome root and soil samples of low-yield forest samples had a significantly higher chao1 index than the high-yield forest samples, indicating that the number of bacterial species in rhizome root and soil of low-yield *P. edulis* was greater than that of high-yield *P. edulis*. This phenomenon was attributed to the enrichment of the special functional bacteria in the rhizosphere of high-yield *P. edulis*, which occupy the niche and lead to fewer bacterial species.

### Beta diversity analysis of the bacterial community

**Figure 7:**
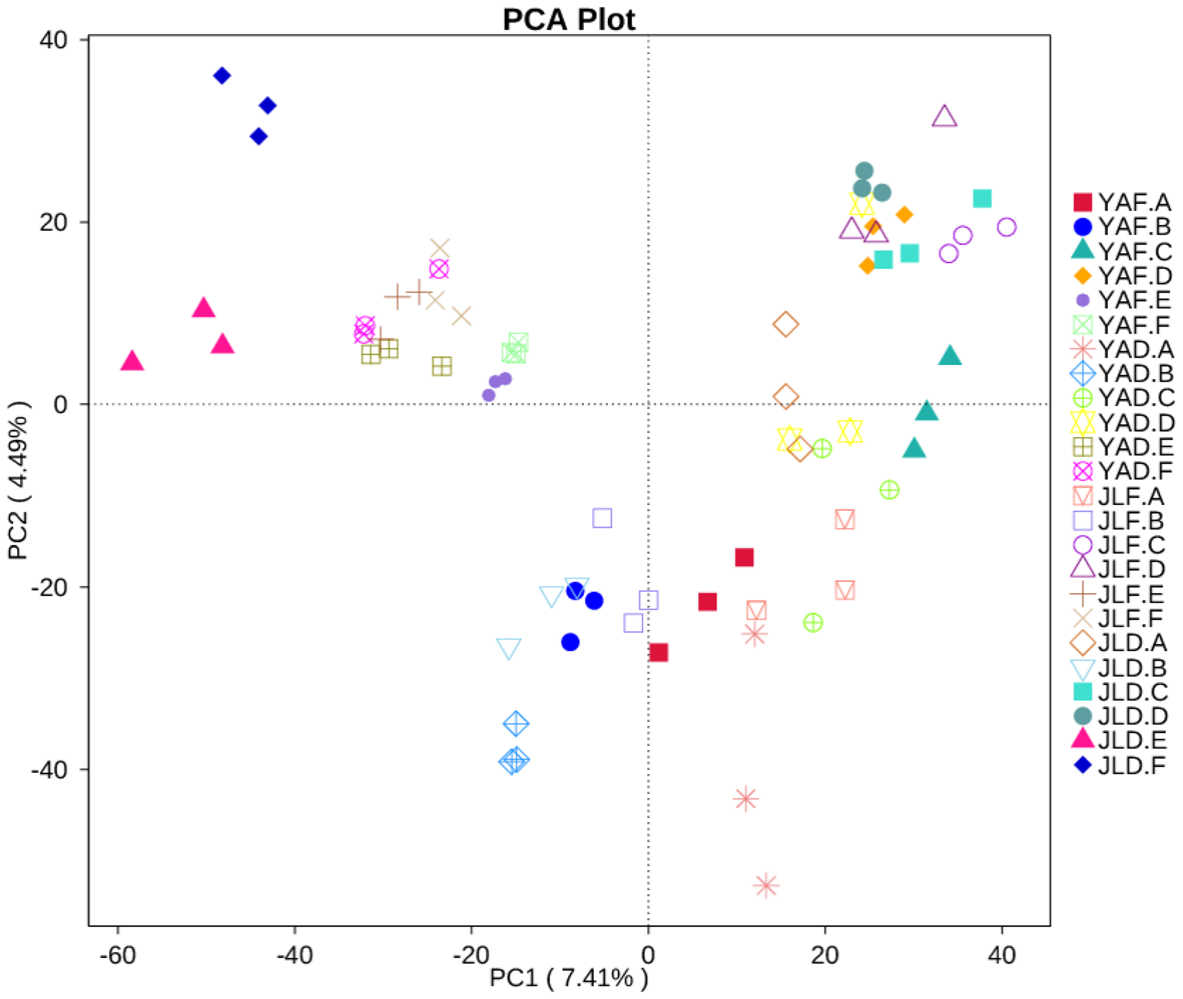
PCA analysis of high-yield and low-yield *P. edulis* forest samples.

**Figure 8:**
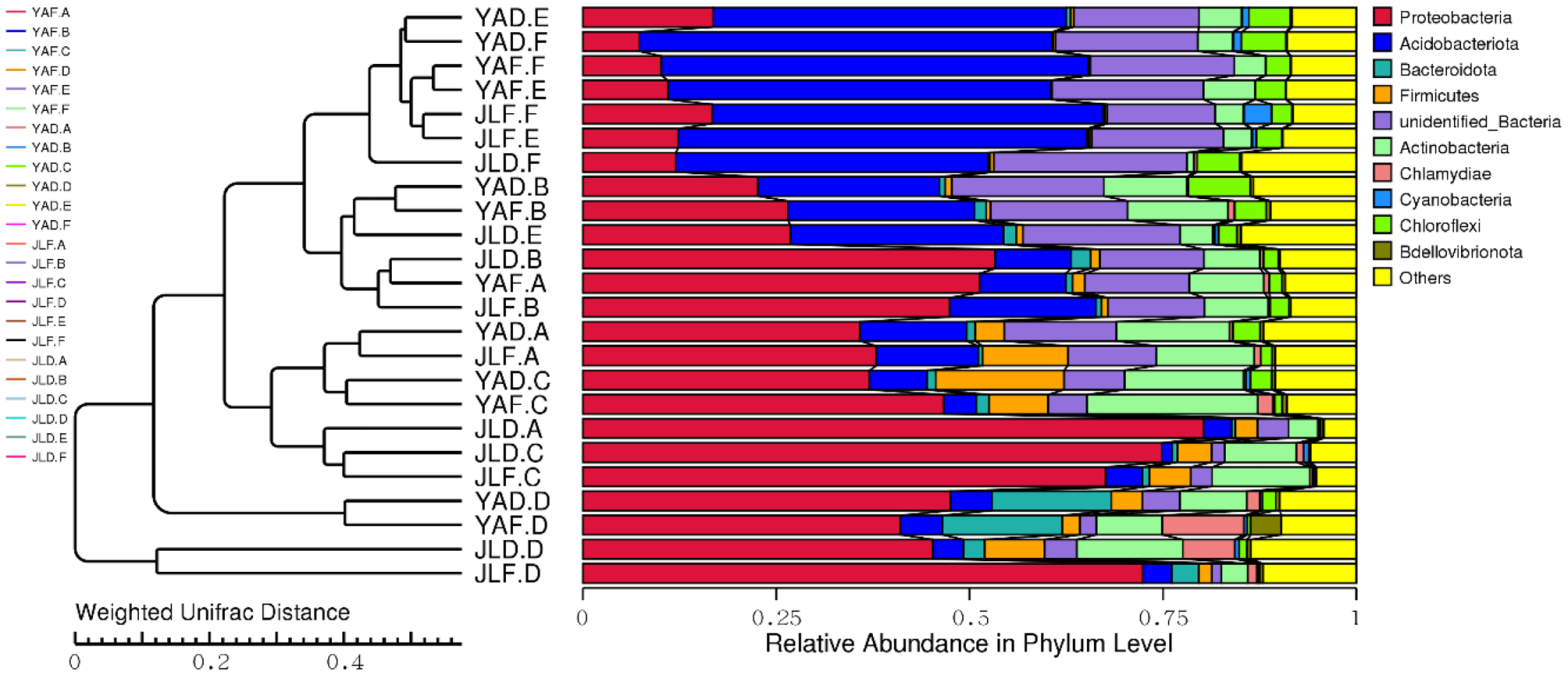
Clustering trees of high-yield and low-yield *P. edulis* forest samples in the two regions.

Figure 7 showed the PCA analysis of each group of samples. The closer the distance between the sample points, the more similar the community composition. Therefore, the similarity and difference of community composition between samples can be preliminarily explored through the distance between sampling points. The clustering tree (Fig. 8) constructed based on the samples showed that the repeated treatments of most samples were clustered in the same or similar groups, indicating that the samples were less disturbed by non-experimental factors.

In Fig. 7, samples of the rhizome and rhizome root of high-yield *P. edulis* in Yong'an and Jiangle are relatively close. In contrast, the rhizome and rhizome root samples of low-yield *P. edulis* were farther apart in the two regions, indicating greater differences between groups. The stem samples of high- and low-yielding *P. edulis* in the same area were relatively close, indicating that the bacterial community composition of *P. edulis* stems (rhizomes) was less affected by high- and low-yields. At the same time, the four groups of bamboo leaf samples were all clustered together, indicating that the bacterial community composition of high- and low-yield *P. edulis* leaves in the two regions had little difference.

In Fig. 8, from the clustering results on the left, the samples can be generally divided into two groups. Compared with other groups, the results of bacterial community in leaves of *P. edulis* in Jiangle were quite different. After further division, the stems of *P. edulis* can be distinguished from the roots and soil samples. It showed that there were differences between the bacterial composition of *P. edulis* stem, roots, and soil samples. The clustering relationship between the low-yielding *P. edulis* forest in Yong'an and the high-yielding rhizome in Jiangle was the closest. The clustering relationship of the high-yielding rhizome root samples of Yong'an *P. edulis* forest and the low-yielding rhizome root samples of Jiangle was the closest. From the relative abundance composition at the sample gate level on the right side of Fig. 8, Proteobacteria accounted for more in the stem and leaf samples of the low-yield *P. edulis* forest, while Acidobacteria mostly appeared in the root and soil samples of the high-yield *P. edulis* forest. In addition, there were some differences in the bacterial community composition of the high- and low-yield forest soil samples in the two regions.

### Prediction of the bacterial functions

**Figure 9:**
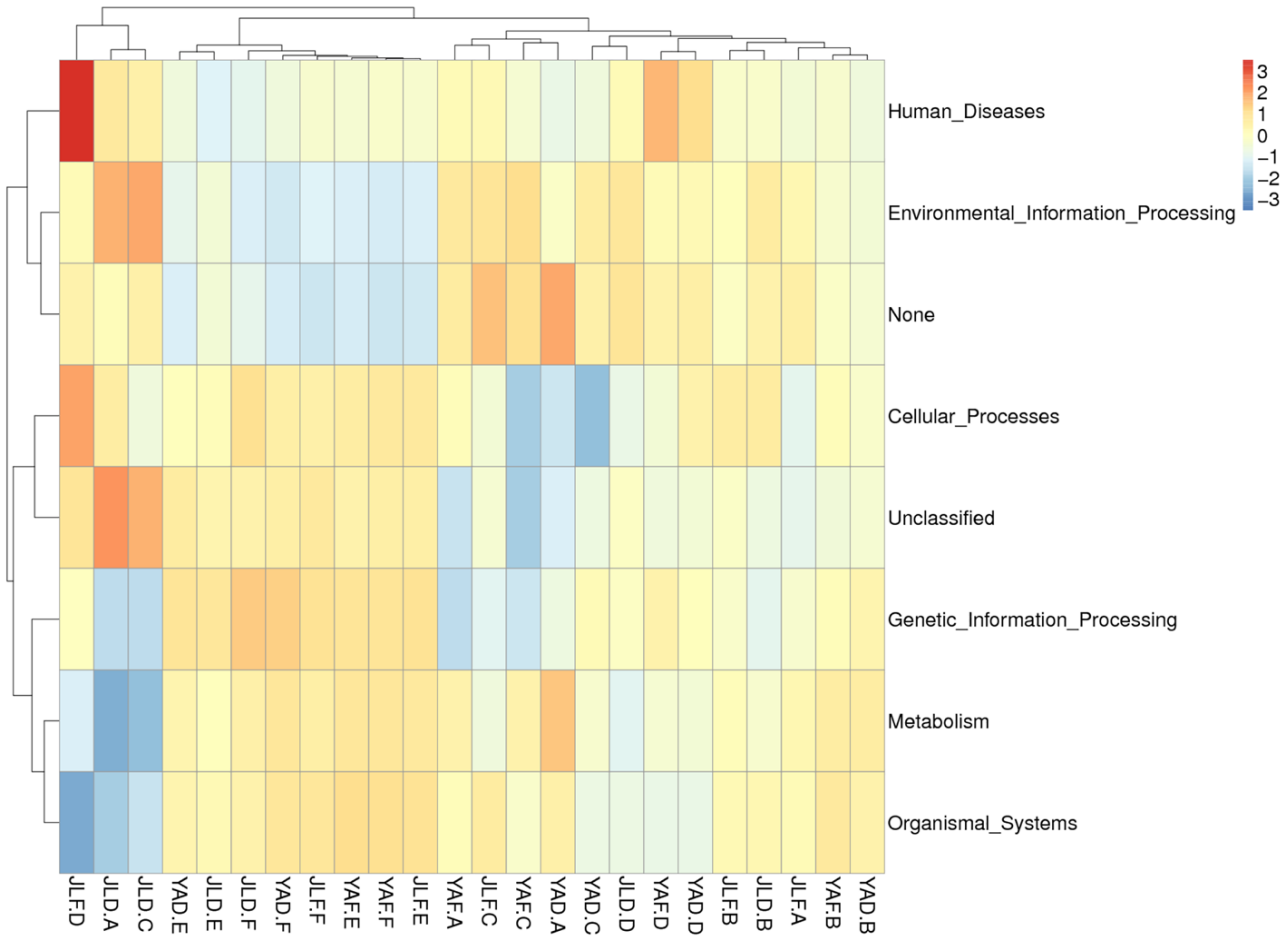
Clustering heat map of PICRUSt functional annotation for high-yield and low-yield *P. edulis* forest samples in the two regions.

Functional prediction analysis was conducted on the high-yield and low-yield *P. edulis* forest samples collected from the two regions. Heat maps were subsequently plotted according to the functional annotation and abundance information of the samples in the database, while clustering was performed from the functional difference level (Fig. 9). The corresponding functions of the bacterial communities included their involvement in organic systems, metabolism, genetic information processing, cellular processes, environmental information processing, and human diseases.

Bamboo stem and leaf samples of the high-yield and low-yield forests in the two regions showed insignificant differences, with no obvious pattern, at the corresponding functional level of the bacterial community. This study hypothesized that the differences partly resulted from occasionality, potentially caused by the environment or other factors, which were not the main factors leading to the high yield and low yield of *P. edulis* forests. However, the soil samples of high-yield and low-yield *P. edulis* forests showed significant differences, exhibiting a reasonable pattern between the two regions. Notably, the abundance of bacterial communities whose corresponding functions were involved in organismal systems, metabolism, genetic information processing, and cellular processes in the soil samples of high-yield *P. edulis* forests was higher than in the soil samples of the low-yield forest. The bamboo rhizome samples of high-yield and low-yield forests in the two regions also showed significant differences but with no obvious pattern. Generally, the differences in the bacterial community functions between high-yield and low-yield *P. edulis* forests were mainly reflected in the bamboo rhizome and soil.

## Discussion

Analysis of endophytic and rhizosphere bacterial community composition found that, the clustering of the high- and low-yield soil bacterial microbial communities in the two regions also had common features. The bacterial community richness of the soil samples from the two places was lower than that of the *P. edulis* rhizome and rhizome root of the same type of samples. The bacterial community richness of soil samples from high-yield forests in both places was lower than that from soil samples from low-yield forests. The number of unique OTUs in rhizome root and rhizosphere soil samples of high-yield *P. edulis* forest was significantly smaller than that of low-yield Moso bamboo forest samples. Moreover, the soil bacterial microbial communities of the high-yield forests in the two places gathered, consistent with the phenomena in poplar^24–26^ and tomato^27,28^. In general, the abundance of rhizome root and rhizosphere soil samples and forest soil samples in high-yielding *P. edulis* forests was low. Studies postulate that the nutrients derived from the exudates and mucus in the rhizosphere of the high-yield forest first attract organisms to the rhizosphere environment, causing the associated bacteria to compete highly for successful colonization^29–32^. This phenomenon necessitates the selection and enrichment of only the special functional bacteria^33^, leading to a decline of bacterial species^21^.

Roots are important tissues for plant growth, development, and reproduction and are the main tissues for the absorption of nutrients and water^34,35^. The underground rhizome root system is the basis for the growth of *P. edulis*. *P. edulis* depends on the system to absorb the nutrients in the soil and supply them to the aboveground part of the plant. The rhizome root system also regulates the nutrient balance between bamboo plants through the bamboo rhizome^3,36^.

This study found there were differences in the bacterial community composition between high-yield and low-yield *P. edulis* forest samples at all classification levels. Some bacterial communities beneficial to soil ecology and nitrogen cycle predominated in high-yield forests. Such as, the relative abundance of Actinobacteria and Acidobacteria of rhizome root samples in the high-yield forest was higher than that in the low-yield forest. Previous studies postulate that Actinobacteria belongs to the eutrophic population, which plays an important role in the carbon cycle^37^ and mainly participates in the decomposition of organic matter^25,38^. Acidobacteria bacteria are important members of soil microorganisms, which play a crucial role in the soil material cycle and ecological environment construction^26,38,39^. The relative abundance of Rhizobiales and Burkholderiales in the bamboo rhizome samples from high-yield *P. edulis* forests was higher than that of samples from the low-yield forest. Rhizobiales function in nitrogen fixation^40^ and participate in the ecosystem's nitrogen repair^27^. Burkholderiales are plant growth-promoting bacteria^41^, which play an important role in biological nitrogen fixation and promoting plant growth^28^. At the genus level, the relative abundance of Bradyrhizobium in bamboo rhizome samples from high-yield *P. edulis* forests in the two regions was higher than that of samples from the low-yield forest. Bradyrhizobium is a group of nitrogen-fixing bacteria widely distributed in soil^42^. They also participate in phosphorus solubilization^29^. From the data of this study, the bacterial community of the underground rhizome root system in the high-yield *P. edulis* forest had a stronger capacity than that in the low-yield forest in the utilization of nutrients, such as carbon, nitrogen, and phosphorus, thereby promoting plant growth and enhancing stress resistance. Gu et al.^43^ reported the existence of a combined nitrogen fixation system in the rhizosphere of *P. edulis* and proposed for the first time that there is a combined nitrogen fixation system in the rhizosphere of *P. edulis*. It indicated that the high-abundance nitrogen-fixing bacteria in the soil of the high-yield *P. edulis* probably also played the role of combined nitrogen fixation. Therefore, the high abundance of these beneficial bacteria indicates that the high and low yield of *P. edulis* may be related to the composition of the bacterial community in the rhizosphere soil and rhizome root system.

This study revealed the differences and patterns of bacterial community composition between high-yield and low-yield *P. edulis* forests. The bacterial community composition of the rhizome root system composed of the bamboo rhizome, rhizome root, and soil was correlated with the high yield and low yield of *P. edulis.* Notably, Actinomycetes, Acidobacterium, and Rhizobium were highly abundant in the high-yield forests. This study provides a basis for the future harnessing and use of microorganisms to enhance the yield of *P. edulis* forests.

## Materials and methods

### Sample collection

Sampling was done in *P. edulis* forest bases in Sanshe village, Xiyang town, Yong'an city, and Wu village, Huangtan town, Jiangle county in Sanming city of Fujian province, China. The sampling area belongs to the subtropical monsoon climate, with geographic coordinates of 117°46′ E, 25°89′ N, and 117°28′ E, 26°43′ N. The bamboo rhizome, rhizome root, stem, leaf, rhizosphere soil, and non-rhizosphere soil of grade II *P. edulis* were collected in the sample plots of high-yield and low-yield forests following the same slope and orientation. The rhizosphere soil was collected the soil which attached to the rhizome roots was brushed with a sterile brush as the rhizosphere soil sample (about 1 mm of soil on the root surface), and the soil was sieved with a 20-mesh sterile sieve to remove obvious plant roots, animal debris. The rhizome, rhizome roots and stems were cut into 10 cm long sections with sterile saws. Each treatment sample was repeated thrice. The samples were placed in sterile sample bags and transported to the laboratory within 24 h for storage at − 20 °C. Table 2 outlines the sample numbers and other relevant information.

**Table 2:** Numbered samples from high-yield and low-yield *P. edulis* forests.

	Yong’an		Jiangle	
	High-yield	Low-yield	High-yield	Low-yield
Rhizome	YAF.A	YAD.A	JLF.A	JLD.A
Rhizome root	YAF.B	YAD.B	JLF.B	JLD.B
Stem	YAF.C	TAD.C	JLF.C	JLD.C
Leaf	YAF.D	YAD.D	JLF.D	JLD.D
Rhizosphere soil	YAF.E	YAD.E	JLF.E	JLD.E
Non-rhizosphere soil	YAF.F	YAD.F	JLF.F	JLD.F

High-yield *P. edulis* forests were defined as forests whose density of standing bamboo was more than 3000 per hectare, the operating density was more than 2250, the average diameter at breast height was more than 9 cm, the yield of bamboo per grade (every 2 years) was more than 12 tons, and the yield of bamboo shoots was more than 3.5 tons. Low-yield *P. edulis* forests were defined as forests whose density of standing bamboo was less than 1800 per hectare, the operating density was less than 1650, the average diameter at breast height was less than 8 cm, the yield of bamboo per grade (every 2 years) was less than 4.5 tons, and the yield of bamboo shoots was less than 1 ton. The classification was based on the “*Technical Regulations for the Cultivation of High-yield P. edulis Forest*” issued by the Fujian Provincial Bureau of Quality and Technical Supervision on October 28, 2011.

### Genomic DNA extraction and high-throughput sequencing on 16S rRNA

The genomic DNA of the samples was extracted using a Tiangen-based kit following the manufacturer’s instructions. DNA quality and quantity were detected through agarose gel electrophoresis, and the DNA samples were stored at − 20 °C until use. Primers were designed based on the conservative and highly variable V5-V7 region to amplify the 16S rRNA gene of bacteria. The primers were 799F (5′-AACMGGATTAGATACCCCKG-3′) and 1391R (5′-GACGGGCGGTGWGTRCA-3′). A sequencing adaptor was added at the end of the primers prior to PCR amplification. PCR products were purified, quantified, and normalized to construct a sequencing library. Quality evaluation for the constructed library was first conducted, after which a small fragment library was built and sequenced on an Illumina NovaSeq sequencing platform using the paired-end sequencing method (Novogene, Beijing).

### Data analysis

Raw sequence data were processed to obtain valid data. OTUs clustering and species information analysis were conducted at a 97% similarity level^44^. A sample dilution curve was then constructed using the R package. A Venn chart and a relative abundance column chart for the species were also created using R after the analysis^45,46^. The Alpha diversity index, Chao 1 and Shannon index, among other indices, were calculated using mothur^47^. Alpha diversity differences between groups were analyzed using the Wilxocon rank-sum test. PCA statistical analysis and plotting were conducted using R. Bacterial populations with significantly different abundance from phyla to genus levels among the different groups were determined through LEfSe analysis (LDA > 4, P < 0.05)^48^. Metabolic functions of bacterial populations were predicted through PICRUSt analysis based on the tree and gene information on OTUs in the database^49^.

### Statements

We have obtained the permission to collect the moso bamboo (*Phyllostachys edulis*) in Sanshe village, Xiyang town, Yong'an city, and Wu village, Huangtan town, Jiangle county in Sanming city of Fujian province, China. The voucher specimens used in this study were deposited in the Mycological Research Center of Fujian Agriculture and Forestry University, Fujian Province, China. The specimens in the center are publicly available, and specimens of the samples are readily available to researchers, the specimen deposit number for this study is 2021-113-5 and identified by Fang Liu. The use of moso bamboo in this study complied with all local, national and international guidelines and regulations. We comply with the IUCN Policy Statement on Research Involving Species at Risk of Extinction and the Convention on the Trade in Endangered Species of Wild Fauna and Flora.

## Data Availability

The original sequences obtained by sequencing have been uploaded to the NCBI SRA database. The relevant accession numbers were PRJNA887215.
